# A systematic review on post-discharge venous thromboembolism prophylaxis in patients with COVID-19

**DOI:** 10.1186/s43044-023-00400-2

**Published:** 2023-08-18

**Authors:** Reza Amani-Beni, Mohammad Kermani-Alghoraishi, Bahar Darouei, Christopher M. Reid

**Affiliations:** 1 Cardiac Rehabilitation Research Center, Cardiovascular Research Institute, Isfahan University of Medical Sciences, Isfahan, Iran; 2 Interventional Cardiology Research Center, Cardiovascular Research Institute, Isfahan University of Medical Sciences, Isfahan, Iran; 3 Hypertension Research Center, Cardiovascular Research Institute, Isfahan University of Medical Sciences, Isfahan, Iran; 4 Centre of Clinical Research and Education, Curtin University, Perth, WA Australia

**Keywords:** Post-discharge, Venous thromboembolism, Thromboprophylaxis, COVID‐19, Systematic review

## Abstract

**Background:**

Coronavirus disease of 2019 (COVID-19) is associated with venous thromboembolism (VTE), not only during hospitalization but also after discharge, raising concerns about anticoagulant (AC) use for post-discharge COVID-19 patients. We aimed to systematically review the current literature on the possible benefits or risks regarding extended thromboprophylaxis.

**Main body:**

We searched related databases from December 1, 2019, to October 6, 2022, including studies on the necessity, duration, and selection of the ideal AC regarding extended thromboprophylaxis for post-discharge COVID-19 patients. The screening of the selected databases led to 18 studies and 19 reviews and guidelines. Studies included 52,927 hospitalized COVID-19 patients, with 19.25% receiving extended thromboprophylaxis. VTE events ranging from 0 to 8.19% (median of 0.7%) occurred in a median follow-up of 49.5 days. All included studies and guidelines, except four studies, recommended post-discharge prophylaxis after an individual risk assessment indicating high thrombotic and low bleeding risk. Studies used risk assessment models (RAMs), clinical evaluation, and laboratory data to identify COVID-19 patients with a high risk of VTE. IMPROVE-DD was the most recommended RAM. Direct oral anticoagulants (DOACs) and low molecular weight heparins (LMWHs) were the most used AC classes.

**Conclusions:**

Post-discharge prophylaxis for COVID-19 patients is recommended after an individual assessment. The IMPROVE-DD model can help predict VTE risk. After distinguishing patients who need post-discharge AC therapy, DOACs for 30–35 days and LMWHs for 40–45 days can be the drug of choice. Further studies, particularly the results of the ongoing randomized controlled trials (RCTs), are required. Also, to properly handle such patients, every physician should consider lifestyle modification in addition to pharmacological treatment for post-discharge VTE prophylaxis.

**Supplementary Information:**

The online version contains supplementary material available at 10.1186/s43044-023-00400-2.

## Background

In December 2019, the Coronavirus disease 2019 (COVID-19) outbreak led to a pandemic [1]. As of November 1, 2022, over 627 million confirmed cases have resulted in more than 6.5 million fatalities worldwide [2]. A significant number of venous thromboembolism (VTE) events have been observed in COVID-19 patients, likely due to endothelium damage, immobility, weakness, and prolonged inflammation [3]. VTE, defined as presenting pulmonary embolism (PE) or deep vein thrombosis (DVT), is a common medical concern associated with potentially fatal complications [4].

Due to different study designs, the prevalence of VTE in hospitalized COVID-19 patients is variable. A previous meta-analysis review, including nearly 2000 COVID-19 patients, reported that the weighted mean prevalence of VTE among Intensive care unit (ICU) and non-ICU patients was 31.3% [5]. In other studies, the VTE pooled prevalence was 17%, with a fourfold higher VTE rate in ICU patients [3, 6]. Due to the reduction in mortality rate and high incidence of VTE in COVID-19 patients, current guidelines recommend using in-hospital thromboprophylaxis for all hospitalized patients, especially critically ill patients [7]. However, even after the disease’s acute phase, patients can still experience VTE after hospital dismissal. In the recent systematic reviews, post-discharge VTE pooled prevalence was reported to be around 1.16–1.8%, suggesting a higher rate than other medically ill patients [8, 9]. 80% of VTE cases occur 30–45 days after hospital discharge [10]. Hence, the appropriate early thromboprophylaxis for COVID-19 discharged patients is essential.

The question to be discussed is the necessity, duration, and selection of the ideal anticoagulant (AC) in post-discharge COVID-19 patients. Several reviews and studies provided evidence regarding the possible benefits of post-discharge AC therapy; for instance, The MICHELLE randomized controlled trial (RCT) studied the necessity and duration of extended thromboprophylaxis using oral ACs [11]. However, as the American Society of Hematology guideline states, studies with a high level of evidence have spoken little about this issue, and the need for systematic review studies to summarize data and provide high-level evidence is required [12]. Furthermore, there are other ongoing RCTs underway, including Post-hospital Thromboprophylaxis RCT (NCT04650087), Hero-19 (NCT04542408), and XACT (NCT04640181), from which no findings have yet been published.

Eventually, still there remains the possibility of COVID-19 pandemic recurrence in the recent future, the spread of new variants, and even similar pandemics [13]. As a result, the question regarding post-discharge thromboprophylaxis in COVID-19 patients remains highly relevant. This practical systematic review seeks to provide a recommendation for physicians based on guidelines, reviews, RCTs, and other current evidence-based data, regarding extended thromboprophylaxis in hospitalized COVID-19 patients without VTE diagnosis at discharge time.

## Main text

### Protocol and registration

This systematic review was reported according to the Preferred Reporting Items for Systematic Reviews and Meta-Analyses (PRISMA) statement and is registered on PROSPERO (Registration Number: CRD42022365107) [14].

### Eligibility criteria

We included peer-reviewed observational studies, RCT studies, and reviews, especially guidelines and position papers reporting the necessity, type, and duration of VTE thromboprophylaxis in post-discharge COVID-19 patients. We excluded conference papers, conference abstracts, erratums, retracted papers, correspondence papers, book titles, and meta-analyses. We also excluded studies carried out on animal or cellular models. Studies had to be available in English.

### Search strategy

We searched PubMed, EMBASE, Web of Science, Scopus, Cochrane, and clinicaltrials.gov from December 1, 2019, to October 6, 2022. We also screened all the review's reference lists by hand-searching. To find relevant literature for the systematic search, we used the search query provided in Additional file 1: Appendix A.1.

### Study selection

We initially screened titles and abstracts of studies for duplication and relevance. The full text of all potentially relevant studies was then independently studied by two authors (R. A. and B. D.) to determine the final study selection. Resolution of disagreement was resolved by consensus and the third author's final decision (M. KA.).

### Data extraction

The following data were extracted by two authors (R. A. and B. D.) from eligible articles: Study characteristics (study titles, authors, year of publication, publication study type, and study site country), population characteristics (number of patients, gender, and age), percentage of patients in the ICU setting, post-discharge thromboprophylaxis name, dosage, and recommended duration of the used AC, risk assessment tool, post-discharge events (thromboembolic events and major bleedings), and duration of follow-ups.

### Risk of bias assessment

Two authors (R. A. and B. D.) assessed the risk of bias and quality of individually selected studies using the Newcastle–Ottawa Quality Assessment Scale (NOS) for cohort studies [15], adapted NOS for cross-sectional studies [16], and the Jadad scale [17] for the RCT studies (Additional file 1: Appendix A.2). NOS and adopted NOS assess the risk of bias within domains, including the study groups' selection, comparability, and the ascertainment of the outcome of interest. The quality of studies was graded using the star system with a maximum possible score of 9 for NOS and 10 for adopted NOS. The Agency for Healthcare Research and Quality (AHRQ) standard was used to convert the NOS (good, fair, and poor) [18]. Thresholds for converting the Adopted NOS (very good, good, satisfactory, and unsatisfactory) were based on a study by Herzog et al. [16]. The Jadad scale evaluated the randomization, blinding, and description of withdrawals with a maximum score of 5. Based on a study by Falagas et al. [19], an RCT with a score of 2 and above was considered a good quality study.

### Data analysis

We used a qualitative analysis and presented the findings with a descriptive approach, odds ratio (OR) with a 95% confidence interval (CI), and risk ratio (RR) with a 95% CI based on the included studies and the summative nature of this systematic review. A meta-analysis and statistical calculations were not performed because the studies' design and reporting differed.

## Results

### Search results

**Fig. 1:**
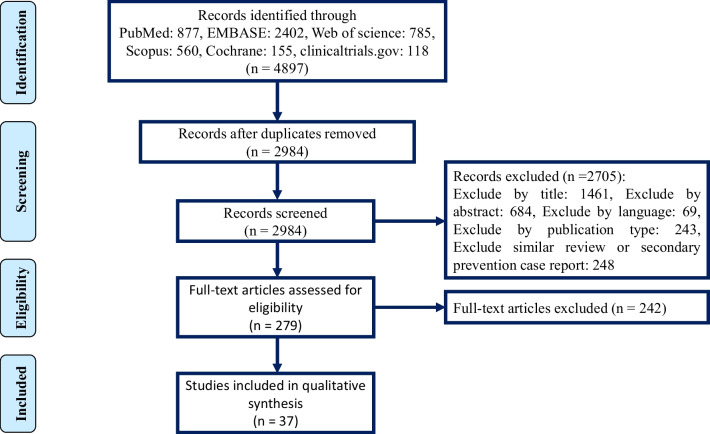
PRISMA flowchart of the literature search and selection of studies that reported about post-discharge thromboprophylaxis

Our initial search in the selected databases yielded 4897 titles, including 37 studies that met the eligibility criteria. The detailed search process is depicted in Fig. 1. 18 out of 37 studies, including eight retrospective cohorts, seven prospective cohorts, two cross-sectional studies, and only one RCT (Table 1). Studies were conducted worldwide, including nine from The United States, two from Russia, and the other six from Norway, Brazil, Spain, Belgium, Singapore, Iran, and England. 19 out of 37 studies were guidelines and reviews, including 14 guidelines, four position papers, and one state-of-the-art review (Table 2). Six Guidelines and reviews were International; the others were from The United States, England, Brazil, Italy, Algeria, Scotland, and Germany.

**Table 1:** Characteristics of included studies reporting on post-discharge thromboprophylaxis

Authors (reference), Year	Study type (Country)	No. of patients (Male %),	Mean or median age (years, [IQR])	Setting % (mean or median days)	Post-discharge AC therapy No (%), type (days)	Risk assessment tool	Post-discharge thromboembolic events (%: DVT%/PE%), Post-discharge Bleeding (%)		Follow-up
							With prophylaxis	Without prophylaxis	
Spyropoulos et al. [28], 2022	Prospective cohort (USA)	37,541	n/a	n/a	7035 (18.74%), LMWH (9.02%), UFH (7.60%), factor Xa inhibitor (6.46%), vitamin K antagonist (1.27%), DTIs (0.21%)	IMPROVE-DD	1007 (2.68%: 1.44/1.24) n/a		70 days
Vaughn et al. [56], 2022	Retrospective Cohort (USA)	523 (49.7%)	68.6 [62–77.8]	ICU 29.6%	–	IMPROVE VTE score ≥ 4 or 2–3 with a D-dimer > 500ng/mL	5 (1%: 0/1), n/a		35 days
Motloch et al. [36], 2022	Retrospective Cohort (Russia)	1002 (43.6%)	59 [48–66]	n/a	1002 (100%), Rivaroxaban (91.6%), apixaban (7.1%), dabigatran (1.3%) (30 days)	n/a	1 (0.1%: 0/0.1), 0 (0%)	–	393 days
		440 (38.9%)	55 [43–63]	n/a	0 (0%)		–	3 (0.7%: 0/0.7), 1 (0.2%)	
Parks et al. [26], 2022	Retrospective cross-sectional (USA)	1121 (53%)	60	ICU 34.17%	38 (3.4%)	n/a	0 (0%), 0 (0%)	5 (0.45%), 4 (0.36%)	30 days
Courtney et al. [30], 2022	Retrospective Cohort (USA)	132 (49.2%)	54	n/a	132 (100%), Rivaroxaban (86.3), enoxaparin (12.9%), apixaban (0.8%) (28 days)	UCHealth post-acute care anticoagulation guidance for COVID-19 inpatients^a^	0 (0%), 1 (0.8%)	–	35 days
		1039 (55.9%)			0 (0%)		–	13 (1.3%: 0.3/1), 1 (0.1%)	
Tholin et al. [57], 2021	Retrospective Cohort (Norway)	223	n/a	n/a	35 (15.7%), LMWH (11.2%), DOACs (4.5%)	Possible risk factors of male and Previous history of VTE	2 (0.9%: 0/0.9), n/a		90 days
Ramacciotti et al. [18], 2021	Multicenter RCT (Brazil)	159 (61%)	57.8	ICU 54%	159 (100%) rivaroxaban 10mg (35 days)	IMPROVE VTE score ≥ 4 or 2–3 with a D-dimer > 500ng/mL	5 (3.17%: 1.89/1.26), 0 (0%)	–	35 days
		159 (59%)	56.4	ICU 50%	0 (0%)		–	13 (8.19%: 2.52/5.67), 0 (0%)	
Quiros Ambel et al. [27], 2021	Prospective cohort (Spain)	95	n/a	n/a	53 (55.79%), LMWH including enoxaparin or bemiparin (2–4 weeks)	PPS	1 (1.05%: 0/1.05), 1 (1.05%)		30 days
Engelen et al. [42], 2021	Prospective Cohort (Belgium)	146 (62%)	58 [51–67]	ICU 39% (13)	41 (28%), enoxaparin (14 days)	Frequent ICU admission, longer hospital/ICU stay, greater need for ventilation, higher D-dimer & CRP level	1 (0.7%: 0/0.7), 0 (0%)	1 (0.7%: 0.7/0), 0 (0%)	6 weeks
Li et al. [22], 2021	Retrospective Cohort (USA)	2832 (52.4%)	63.4 [53–75]	ICU 15.18%	188 (6.64%), prophylaxis dose of Apixaban, Enoxaparin, and Rivaroxaban 494 (17.44%), therapeutic dose of Apixaban, Enoxaparin, Rivaroxaban, Dabigatran, Edoxaban, Warfarin	History of VTE, pre-discharge level CRP > 10mg/dl, peak D-dimer levels > 3μg/mL	2 (0.07%), n/a	34 (1.2%), n/a	90 days
Tan et al. [20]. 2021	Prospective cohort (Singapore)	63	40	n/a	0 (0%)	IMPROVE	–	0 (0%: 0/0), n/a	6–8 weeks
Stawiarski et al. [23], 2021	Cross-sectional study (USA)	91	n/a	n/a	7 (7.69%), enoxaparin	Elevated D-dimer, family history of DVT, prolonged hypoxic respiratory failure with suspected possible PE	0 (0%), 1 (1.09%)	1 (1.09%: 0/1.09), 0 (0%)	90 days
Giannis et al. [47], 2021	Prospective Cohort (USA)	4906 (53.7%)	61.7 [36.7–86.7]	ICU 11.8%	581 (11.84%), Prophylactic dose of Enoxaparin (1.3%), UFH (0.06%), Apixaban (3.7%), and Rivaroxaban (6.9%) 31 (0.63%), Therapeutic dose of Enoxaparin (0.3%), Rivaroxaban (0.02%), Warfarin (0.4%)	IMPROVE-DD score ≥ 4, advanced age, cardiovascular risk factors, Chronic kidney disease, and ICU stay	76 (1.55%: 0.9/0.85), 85 (1.73%)		92 days
Tsaplin et al. [40], 2021	Retrospective Cohort (Russia)	151	n/a	n/a	13 (8%), AC (12 days)	Caprini score	0 (0%), 0 (0%)	1 (0.7%: 0.7/0), 1 (0.7%)	6 months
Eswaran et al. [25], 2021	Retrospective cohort (USA)	447 (51.5%)	54.4	ICU 39.4%,	190 (42.5%), DOAC (38.25%) especially Rivaroxaban and Apixaban (30 days)	n/a	3 (0.67%: 0/0.67), n/a		30 days
Rashidi et al. [24]. 2020	Prospective Cohort (Iran)	1529 (54.4%)	56	ICU 7.8%	71 (4.6%)	n/a	3 (0.2%:0/0.2) n/a		45–55 days
Salisbury et al. [33], 2020	Prospective Cohort (England)	152	61.5 [52–75]	ICU 16%	5 (3%), LMWH (7 days)	n/a	4 (2.6%: 0/2.6), 0 (0%)		42 days
Patell et al. [10], 2020	Retrospective cohort (USA)	163 (47.8%)	58 [44–67]	ICU 26%	0 (0%)	n/a	–	1 (0.6%: 0/0.6), 6 (3.7%)	30 days
		13	n/a	n/a	13 (100%), LMWH (76.92%), DOAC (15.38%), UFH (7.69%), (2 weeks for pregnant patients)	Pregnancy, orthopedic procedure, COVID-19, coagulopathy	0 (0%: 0/0), 0 (0%)	–	

*IQR* interquartile range, *AC* anticoagulation, *DVT* deep venous thrombosis, *PE* pulmonary embolism, *n/a* not available, *LMWH* low molecular weight heparin, *UFH* unfractionated heparin, *DTI* direct thrombin inhibitor, *IMPROVE-DD* International Medical Prevention Registry on Venous Thromboembolism and D-dimer, *ICU* intensive care unit, *COVID-19* coronavirus disease of 2019, *DOAC* direct oral anticoagulants, *VTE* venous thromboembolism, *PPS* padua prediction score, *CRP* C-reactive protein

^a^IMPROVE risk score ≥ 4, patients with VTE risk factors (e.g., active cancer, pregnancy, comorbid chronic inflammatory or autoimmune condition), patients who received therapeutic anticoagulation for “hyper inflammatory state” without clinical suspicion of VTE or thrombosis, patients who received intensified prophylaxis during hospitalization (recommended for patients with a D-dimer > 1500)

**Table 2:** Characteristics of guidelines and reviews reporting on post-discharge thromboprophylaxis

Name (reference), last update	Study type (Country)	Risk assessment tool	Post-discharge AC therapy recommendation	Post-discharge AC therapy type (days)
NIH^a^ [34], September, 2022	Guideline (USA)	n/a	Recommends against routinely continuing post-discharge VTE prophylaxis Consider post-discharge thromboprophylaxis after an individualized risk assessment for patients with high VTE risk and a low bleeding risk	n/a
NICE^b^ [21], July, 2022	Guideline (England)	COVID-19 patients who need low-flow or high-flow oxygen, continuous positive airway pressure, non-invasive ventilation or invasive mechanical ventilation, and who do not have an increased bleeding risk	Recommends In-hospital thromboprophylaxis continue in young people and adults with COVID-19 with risk factors for 7 days, including after discharge	Standard prophylactic dose of LMWH
ISTH^c^ [50], July, 2022	Guideline (International)	IMPROVE score of ≥ 4 or 2–3 with a D-dimer above the upper limit of normal, and without contraindication (e.g., high risk of bleeding, pregnancy, lactation)	Recommends in patients with VTE risk factors and without contraindications	Rivaroxaban 10mg daily (30 days)
ASH^d^ [12]. May, 2022	Guideline (USA)	n/a	Recommends against routinely continuing post-discharge VTE prophylaxis Consider post-discharge thromboprophylaxis after an individualized risk assessment for patients with high VTE risk and a low bleeding risk	n/a
The Anticoagulation Forum [35], March, 2022	Guideline (USA)	IMPROVE VTE score ≥ 4 or score 2–3 with elevated D-dimer and not at increased risk of bleeding regardless of the intensity of their inpatient thromboprophylaxis	Recommends against routinely continuing post-discharge VTE prophylaxis Consider post-discharge thromboprophylaxis after an individualized risk assessment for patients with high VTE risk and a low bleeding risk	Rivaroxaban 10mg daily (35 days)
ESCMID^e^ [45], February, 2022	Guideline (International)	Active malignancy, immobility, history of VTE, recent major surgery, thrombophilia	Recommends post-discharge thromboprophylaxis after an individualized risk assessment for patients with high VTE risk and a low bleeding risk	n/a
Brazilian Guideline [58], November, 2021	Guideline (Brazil)	Patients with specific clinical indications (e.g., atrial fibrillation and VTE). RAMs such as PPS and IMPROVE may be used as support	Recommends against routinely continuing post-discharge VTE prophylaxis The indication for the use of AC after discharge should follow the same criteria applied for non-COVID-19 patients	n/a
Italian Guideline^f^ [59]. October, 2020	Guideline (Italy)	PPS ≥ 4 or IMPROVE score ≥ 4 and low bleeding risk	Recommends post-discharge thromboprophylaxis after an individualized risk assessment for patients with high VTE risk and a low bleeding risk	(45 days)
SATH^g^ [43]. October, 2020	Guideline (Algeria)	Prolonged immobilization, age > 70 years, history of VTE, comorbidity (e.g. cancers), D-dimer > 2 times the normal rate (threshold adjusted according to age)	Recommends post-discharge thromboprophylaxis after an individualized risk assessment for patients with high VTE risk and a low bleeding risk	LMWHs at prophylactic doses or DOACs (45 days)
VAS^h^ [52], July, 2020	Position paper (International)	IMPROVE-DD RAM	Recommends post-discharge thromboprophylaxis in patients at high VTE risk after discharge with creatinine clearance ≥ 30mL/min	Rivaroxaban 10mg or Betrixaban 80mg daily or prophylactic weight-adjusted doses of LMWH (40 days)
SIGN^i^ [60], July, 2020	Guideline (Scotland)	IMPROVE VTE RAM	Recommends post-discharge thromboprophylaxis after an individualized risk assessment for patients with high VTE risk, low bleeding risk, and no known contraindications	LMWHs or DOACs (2 weeks)
BSTH and ABHH^j^ [48]. June, 2020	Position paper (Brazil)	Age > 75 years, previous history of VTE, known thrombophilia, active cancer, obesity, use of estrogen, or chronic heart or respiratory failure	Recommends for high risk patients or those who maintain immobility if there is no contraindication after reevaluation. In case of early discharge consider pharmacological thromboprophylaxis for at least 7 days	n/a
DGA^k^ [49]. June, 2020	Position paper (Germany)	previous VTE, active cancer, high-risk thrombophilia, BMI > 35 kg/m^2^	Recommends post-discharge thromboprophylaxis after an individualized risk assessment for patients with high VTE risk and a low bleeding risk	(at least 1–2 weeks)
CHEST^l^ [61]. June, 2020	Guideline (USA)	n/a	Recommends post-discharge thromboprophylaxis after an individualized risk assessment for patients with high VTE risk and a low bleeding risk	n/a
SSC-ISTH^m^ [44]. May, 2020	Guideline (International)	Advanced age, ICU stay, cancer, previous history of VTE, thrombophilia, severe immobility, elevated D-dimer (> 2 times), IMPROVE ≥ 4	Recommends for all hospitalized patients with COVID-19 with high risk of VTE	LMWH, DOAC (i.e., rivaroxaban or betrixaban)
Health System Anticoagulation Task Force [51], May, 2020	Guideline (USA)	IMPROVE-DD RAM	Recommends post-discharge thromboprophylaxis after an individualized risk assessment for patients with high VTE risk and a low bleeding risk. The patients should have a creatinine clearance and liver function panel in addition to platelet count (> 25,000 mm^3^) prior to the initiation of extended prophylaxis	Rivaroxaban 10mg daily or as an alternative, Enoxaparin 40mg Qday subcutaneously if the CrCl ≥ 30ml/min (6 weeks)
Global COVID-19 Thrombosis [41]. April, 2020	JACC State-of-the-Art Review (International)	Caprini, IMPROVE and PPS as RAM. Elevated risk of VTE (e.g., reduced mobility, comorbidities such as active cancer, and elevated D-dimer > 2 times the upper limit of normal) with low risk of bleeding	Recommends post-discharge thromboprophylaxis after an individualized risk assessment for patients with high VTE risk and a low bleeding risk	LMWH or DOACS (up to 45 days)
Chinese Guideline^n^ [62], April, 2020	Guideline (International)	n/a	Mild and moderate COVID-19 patients perceived to have a persistent risk of VTE at the time of discharge, a prolonged out-patient VTE prophylaxis care should be considered	LMWH over DOAC
SISET^o^ [46]. April, 2020	Position paper (Italy)	pre-existing or persisting VTE risk factors: reduced mobility, BMI > 30, previous VTE, active cancer	Recommends post-discharge thromboprophylaxis in case of pre-existing or persisting VTE risk factors	(7–14 days)

*AC* anticoagulant, *n/a* not available, *VTE* venous thromboembolism, *COVID-19* coronavirus disease of 2019, *LMWH* low molecular weight heparin, *IMPROVE* International Medical Prevention Registry on Venous Thromboembolism, *RAM* risk assessment model, *PPS* padua prediction score, *DOAC* direct oral anticoagulants, *IMPROVE-DD* IMPROVE and D-dimer, *BMI* body mass index, *ICU* intensive care unit

^a^National Institutes of Health

^b^National Institute for Health and Care Excellence

^c^International Society on Thrombosis and Haemostasis

^d^The American Society of Hematology

^e^European Society of Clinical Microbiology and Infectious Diseases

^f^Italian Working Group on Atherosclerosis, Thrombosis and Vascular Biology

^g^Algerian society of transfusion and hemobiology

^h^VAS-European Independent Foundation in Angiology/Vascular Medicine

^i^Scottish Intercollegiate Guidelines Network

^j^Brazilian Society of Thrombosis and Hemostasis and the Thrombosis and Hemostasis Committee of the Brazilian Association of Hematology, Hemotherapy and Cellular Therapy

^k^German Society of Angiology

^l^American College of Chest Physicians

^m^Scientific and Standardization Committee of the International Society of Thrombosis and Hemostasis

^n^Chinese Consensus Statement Group

^o^Italian Society on Thrombosis and Hemostasis

### Risk of bias assessment

The systematic review included 18 studies. Four cohorts were of good quality, and the other 11 were studies with lower quality scores, mainly due to comparability issues. One of the cross-sectional studies was of good quality, and the other was of satisfactory quality. The RCT was of good quality. The majority of included studies (12/18) were of low quality, and the others (6/18) were of high quality (Additional file 1: Appendix A.2).

### Characteristics of patients and included studies

The major characteristics of included studies are summarized in Table 1. This systematic review included 18 studies with a total of 52,927 patients. Thirteen studies reported the mean age ranging from 40 to 68.6 years. The follow-up period of the included studies was different, ranging from 30 to 393 days after hospital discharge (median of 49.5 days). All the studies reported the rate of thromboembolic events in their follow-up duration, with a total of 1182 VTE events ranging from 0 to 8.19% (median of 0.7%). All but three studies reported that the PE ratio is equal to or greater than DVT. Eight studies reported the rate of post-discharge bleeding ranging from 0 to 3.7%. (median of 0%). Only one study did not use ACs after hospital discharge for any patients, and the others used AC for a total of 10,088 patients (19.25% of all) [20]. Ten studies reported the rate of ICU patients ranging from 7.8 to 54%, where the highest rate of ICU patients was in the MICHELLE study with the highest ratio of post-discharge VTE events [11]. Also, the major characteristics of included guidelines and reviews are summarized in Table 2.

### Post-discharge thromboprophylaxis: necessity, evaluation, and AC selection

Based on the results of the included guidelines and studies, there are controversial views on post-discharge thromboprophylaxis. All the guidelines but one [21], and most studies (11/18), were in favor of this matter, but after an individual risk assessment; indicated in post-discharge COVID-19 patients with high VTE risk, low bleeding risk, and no known contraindications (Tables 1 and 2). Li et al. reported a reduced risk post-discharge VTE in patients who received the therapeutic AC at discharge (OR: 0.18 and 95% CI 0.04–0.75); however, the association of post-discharge prophylactic AC with post-discharge VTE was insignificant [22]. As the only published good-quality RCT, the MICHELLE trial study investigated the VTE and bleeding outcomes in the rivaroxaban and control group at day 35. Post-discharge thromboprophylaxis reduced the risk of VTE events by 67% in the rivaroxaban group (95% CI 0.12–0.90), and no major bleeding occurred [11]. Nevertheless, three cohorts and one cross-sectional study implied no role for post-discharge thromboprophylaxis [20, 23–25]. Additionally, three cohort studies did not provide a definite opinion on this matter [26–28].

Studies used risk assessment models (RAMs), clinical evaluation, and laboratory data to identify COVID-19 patients with high post-discharge thrombotic risk. Almost half of the guidelines (9/19) used RAMs which all mentioned The International Medical Prevention Registry on Venous Thromboembolism (IMPROVE). IMPROVE is a RAM consisting of seven variables, including the previous episode of VTE (3 points), known thrombophilia (2 points), current paralysis or paresis of lower-limb extremity (2 points), Current cancer (2 points), ICU/CCU stay (1 point), immobilization (1 point), and age > 60 years (1 point), categorizing COVID-19 patients into low (0–1 score), moderate (2–3 score), and high VTE risk (≥ 4 scores) [29]. Two high-quality studies, including Courtney et al. and Ramacciotti et al., reported a significant association between a higher IMPROVE VTE risk score and receiving extended thromboprophylaxis [11, 30]. Accordingly, in the MICHELLE trial study, with increasing the modified IMPROVE VTE risk score from 2–3 to ≥ 4, the RR increased by 27% in a way that patients with IMPROVE VTE score ≥ 4 or 2–3 with a D-dimer > 500 ng/mL were suitable for receiving extended thromboprophylaxis [11]. The IMPROVE-DD RAM with eight variables, including the D-dimer (2 points), has a similar cut-off as IMPROVE for high-risk VTE patients, [31]. In a prospective cohort CORE-19 registry, Giannis et al. demonstrated that the IMPROVE-DD RAM score ≥ 4 was significantly associated with an increased risk of VTE, arterial thromboembolism, and mortality in post-discharge COVID-19 patients (OR: 3.64 with 95% CI 2.91–4.55) [32]. Padua Prediction Score (PPS) (4/37) and the Caprini model (2/37) were used less in the included studies. Moreover, most included studies (6/18) and guidelines (10/19) used clinical evaluation as an important factor for assessing the VTE risk. 6/18 studies and 5/19 guidelines mentioned lab data, especially D-dimer (Tables 1 and 2).

Direct oral anticoagulants (DOACs) and low molecular weight heparins (LMWHs) have been used more than other AC classes in the reviewed studies, with 9/18 included studies and 8/19 guidelines suggesting LMWH; 8/18 included studies, and 9/19 guidelines suggesting DOACs. Unfractionated heparin (UFH) and vitamin K antagonist both with 3/18 included studies but none of the guidelines mentioned any of these two AC classes. Cohort studies have reported a post-discharge thromboprophylaxis of 7–28 days for LMWHs and 30–35 days for DOACs, while in guideline studies, the range is between 14 and 45 days for both AC classes (Tables 1 and 2).

## Discussion

**Fig. 2:**
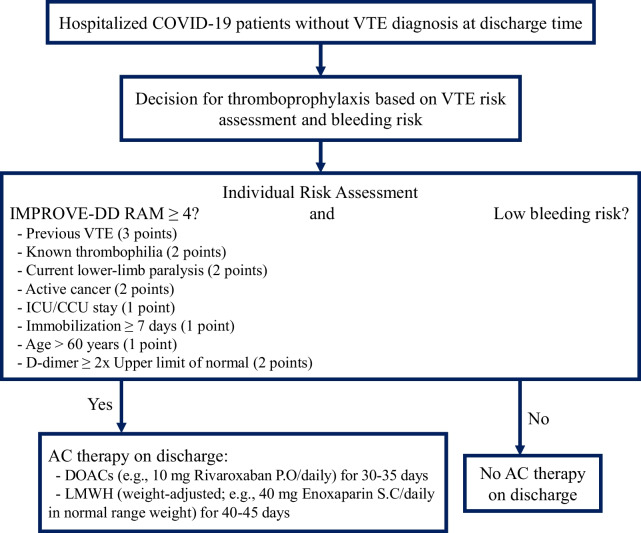
Suggested algorithm for post-discharge thromboprophylaxis in COVID-19 patients. COVID-19 = coronavirus disease of 2019; VTE = venous thromboembolism; IMPROVE-DD = International Medical Prevention Registry on Venous Thromboembolism and D-dimer; ICU = intensive care unit; CCU = cardiac care unit; AC = anticoagulant; DOAC = direct oral anticoagulants; P.O = per os; LMWH = low molecular weight heparin; S.C = subcutaneous

COVID-19 disease seems to be associated with a higher risk of VTE incidence, especially in more severe cases [8]. This practical systematic review aimed to determine the need to receive thromboprophylaxis and whether post-discharge thromboprophylaxis improves outcomes, including decreasing VTE events accompanying low bleeding risks. Then, identifying high-risk VTE patients and post-discharge thromboprophylaxis management, including the type of drug, dosage, and medication duration, will be discussed. Figure 2 provides a pragmatic approach for managing post-discharge thromboprophylaxis in COVID-19 patients without VTE diagnosis at discharge time based on the available evidence.

### Should COVID-19 patients receive post-discharge VTE thromboprophylaxis?

While several observational cohort studies, RCTs, and guidelines studied thromboprophylaxis during and after hospitalization, the role of post-discharge VTE thromboprophylaxis remains controversial [12, 22, 33, 34]. Most guidelines recommend against routinely continuing VTE prophylaxis after hospital discharge [34, 35]. Still, they suggest an individual risk assessment and using ACs after discharge in patients with high thrombotic risk, low bleeding risk, and no contraindications (Table 2).

Likewise, most of the included studies in this systematic review agreed with post-discharge VTE thromboprophylaxis if the patient's risk assessment indicated a high-risk situation for VTE. In between, three good-quality cohort studies reported a significant association between post-discharge VTE risk reduction and extended thromboprophylaxis [22, 30, 36]. As in the study by Li et al., this risk reduction was stated to be 82%; although, in the Courtney et al. study, the chance of bleeding increased significantly with post-discharge AC [22, 30]. The MICHELLE trial provided valuable information about post-discharge VTE thromboprophylaxis. The results showed that AC therapy in high-risk patients after discharge reduces the VTE events, and the risk of bleeding will remain unchanged [11].

Three cohort studies and one cross-sectional study suggested against using extended thromboprophylaxis due to their results that only the Eswaran et al. study was of Good quality and the others were of studies with lower quality scores [20, 23, 25, 37]. It is worth saying that these four studies had the lowest average age among the included studies. Tan et al. included patients with few comorbidities and the IMPROVE VTE score of 0 or 1 in 91.3% of all patients [20]. Stawiarski et al. evaluated patients with low D-dimer levels and moderate COVID-19 disease [23]. These three poor-quality studies had few ICU-admitted patients, which has been proven important in increasing the risk of VTE after discharge [20, 23, 37]. The Eswaran et al. study found no correlation even after adjusting for possible confounders such as age and ICU admission [25]. This matter can be attributed to the lack of accurate follow-up and AC thromboprophylaxis in high-risk patients, which may have led to a low incidence of VTE.

#### Recommendation

Due to the inflammatory state and the chance of post-discharge recurrence of VTE in COVID-19 patients, we suggest that the physicians decide on extended thromboprophylaxis based on individual assessment of VTE and bleeding risk.

### Which COVID-19 patients should receive post-discharge thromboprophylaxis? Tools, lab data, and clinical evaluation

Predicting VTE risk, identifying hospitalized patients with COVID-19 at high VTE risk, and discriminating who may benefit from post-discharge thromboprophylaxis with a low risk of major bleeding remains a critical clinical issue [38]. Several tools and models, including the Caprini model, the IMPROVE VTE RAM, the modified IMPROVE RAM, the IMPROVE-DD RAM, the PPS, and the Wells model have been used in COVID-19 patients to assess the need for thromboprophylaxis. IMPROVE RAM was the most applied RAM among the studies to assess the VTE risk in post-discharge COVID-19 patients, and the other RAMs were less used by studies or recommended by guidelines. In a study by Goldin et al. in 9407 patients, the IMPROVE VTE RAM without D-dimer demonstrated a sensitivity of 83.9% and specificity of 29.2% [31]. MICHELLE RCT used modified IMPROVE RAM assigned to COVID-19 patients with IMPROVE score of ≥ 4 or 2–3 with an elevated D-dimer (> 2 times the upper limit of normal or as stated in MICHELE RCT with a D-dimer > 500 ng/mL) for patients with increased risk of VTE [11, 39]. For this reason, IMPROVE-DD eliminates the need for separate grouping using a D-dimer and increases validity scores to a sensitivity of 97.1% and specificity of 21.5% simultaneously [31]. Furthermore, various guidelines have also suggested the IMPROVE RAM, which is either the IMPROVE-VTE RAM with D-dimer or IMPROVE-DD itself (Table 2).

Tsaplin et al. [40] used the original Caprini score (2005 version) and eight modified versions to predict VTE frequency. Among the four modifications used to predict the risk of symptomatic VTE 6 months after discharge, all the versions demonstrated high sensitivity and specificity, especially Caprini with D-dimer and Caprini with COVID-19 risk scores with a sensitivity of 75% and a specificity of 81%. However, the original Caprini score correlates significantly with the VTE risk with the cut-off score of seven [40]. More studies are needed to evaluate the modified versions of the Caprini score. A retrospective cohort study also validated Caprini and IMPROVE RAM as a practical RAM independent of each other [39].

Not all VTE risk assessments are based on models and scores but on the patient's lab data and clinical evaluations. Lab data including D-dimers > two times upper the normal limit (threshold adjusted according to age) [11, 23, 30, 41–44], and pre-discharge C-reactive protein (CRP) level > 10mg/dl [22, 42] are important factors having significant association with increasing the risk of VTE [33]. In this regard, Li et al. reported a 3.76-fold (95% CI 1.86–7.57) and 3.02-fold (95% CI 1.45–6.29) higher risk of VTE with patient's peak D-dimer levels greater than 3μg/mL and pre-discharge CRP levels greater than 10mg/dL, respectively [22].

Clinical evaluations have long been essential, with easy access to assess the thrombosis risk. Prolonged immobilization [41, 43–46], advanced age (> 70–75 years) [43, 44, 47, 48], previous history of VTE [22, 43–46, 48, 49], active cancer [30, 41, 43–46, 48, 49], known thrombophilia [44, 45, 48, 49], and chronic heart or respiratory failure [21, 23, 47, 48] are the most important factors increasing the VTE risk that will be examined during the clinical evaluation. Some clinical risk factors are not included in IMPROVE-DD RAM. However, they are mentioned in the included studies, including obesity, use of estrogen, family history of VTE**, **comorbid chronic inflammatory or autoimmune condition, chronic kidney disease (CKD)**, **recent major surgery (e.g., orthopedic procedure), and atrial fibrillation (Tables 1 and 2). Pregnancy is a controversial indication; two included studies reported pregnancy as an indication [10, 30], while the ISTH guideline [50], due to the risk of bleeding, has reported it as a contraindication, demonstrating greater consideration during the risk of bias assessment.

#### Recommendation

Clinical evaluation and laboratory data are practical factors in AC thromboprophylaxis. The most important clinical risk factors are prolonged immobilization, advanced age, previous history of VTE, active cancer, known thrombophilia, and chronic heart or respiratory failure. In this regard, IMPROVE-DD RAM is designed based on most of the mentioned risk factors and has shown good efficiency in assessing high-risk VTE events in COVID-19 patients without VTE diagnosis at discharge time.

### Post-discharge VTE AC thromboprophylaxis in patients with COVID-19: which and how?

The choice of medications, dosing, and duration of thromboprophylaxis should be based on high-quality, evidence-based data and guideline recommendations. Recommended drug medication to prevent thrombosis can be placed in four popular classes of ACs, including LMWHs, DOACs, UFH, and vitamin K antagonists. Several studies recommended DOACs as a post-discharge thromboprophylaxis agent. Three high-quality studies, including the MICHELLE trial, recommended rivaroxaban 10mg daily for 30–35 days. Alternatively, apixaban 2.5mg BID and dabigatran 110mg BID can be used as the choices of DOACs [11, 25, 36]. Also, ISTH, the anticoagulation forum, the VAS, and the health system anticoagulation task force guidelines favored rivaroxaban 10mg daily for 30–42 days [35, 50–52]. The VAS guideline also recommended betrixaban 80mg daily for 40 days [52].

Several cohort and guideline studies recommended LMWHs, especially enoxaparin. In this regard, Quiros Ambel et al. provided a protocol in which patients in the absence of hemorrhagic risk and high risk of thrombosis should receive weight or albumin/creatinine ratio (ACR) adjusted LMWH (enoxaparin or bemiparine) for 4–6 weeks [27]. Patients weighted ≤ 50 kg or elderly patients with ACR < 30 ml/min should receive 2500 IU sc/day of bemiparine or 20mg sc/day of enoxaparin, patients weighted 51–80 kg should receive 40mg sc/day of enoxaparin or 3500IU sc/day of bemiparine. Finally, patients who weighed 81-100 kg and > 100 kg were suggested to receive 60mg sc/day of enoxaparin and 80mg sc/day of enoxaparin, respectively. In the same direction, Engelen et al. suggested enoxaparin 0.5 mg/kg daily for 14 days, and Giannis et al. used any dose of enoxaparin < 80 mg daily [42, 47]. In addition, the health system anticoagulation task force guideline recommends enoxaparin 40mg Qday subcutaneously for 6 weeks as an alternative over DOACs [51]. Generally, apart from Li et al., all other included studies emphasize the preference for prophylactic dosage over therapeutic dosage [22]. Regarding the selection of the recommended duration for extended prophylaxis, the included studies have suggested a shorter duration than the guidelines [33, 42, 47]. However, the majority of the guidelines have suggested 40–45 days [41, 43, 51, 52]. Finally, due to limitations, such as INR checks for warfarin and the need for injection for UFH and fondaparinux, the two classes of drugs, LMWH and DOACs, seem to be more acceptable.

#### Recommendation

If a COVID-19 patient needs extended thromboprophylaxis, we suggest oral AC medications such as DOACs, especially rivaroxaban 10mg daily for 30–35 days, and subcutaneous AC drugs such as the LMWH family, especially weight-adjusted enoxaparin, for 40–45 days. Depending on the specialist's evaluation and the persistence of VTE risk factors, an individual risk assessment should be repeated, and, if necessary, the length of thromboprophylaxis should be continued.

### Role of lifestyle modification

The immune system and hemostasis have a close relationship, with each system protecting the host and preventing the spread of foreign diseases [53]. In patients with COVID-19, immunothrombosis has been hypothesized as a pathogenic mechanism in which endothelial dysfunction, hypercoagulability, and activation of innate immune cells contribute to the observed prothrombotic condition [54]. In addition, several environmental factors can affect a person's immune system. In order to have a healthy lifestyle and thus a better immunity system, we can refer to [E(e)SEEDi], which includes five fundamental items: "External and internal environment—Sleep—Emotion—Exercise—Diet" Interventions, also known as magic polypill [55].

Modifications such as communication with loved ones, washing hands, 7–9 h of sleep at night, control of obstructive sleep apnea, decreasing anxiety and depression, maintaining a healthy weight by exercise, anti-inflammatory/antioxidant diet, quitting smoking and reducing alcohol consumption are beneficial E(e)SEEDi for every COVID-19 patients [55].

Cardiovascular events, including VTE, are closely related to a person's lifestyle, and E(e)SEED imbalance can reduce the body's immunity and, as a result, increase the risk of cardiovascular events. In this regard, in addition to pharmacological treatment in post-discharge VTE prophylaxis, every physician should consider lifestyle modification to manage such patients thoroughly [55].

## Limitation

The limitations of this study include the use of only one published RCT and other related clinical trial studies are ongoing and have not yet been published. For this reason, most of the data presented in this practical systematic review are from cohort studies and guidelines. Due to the rapid rate of newly published articles on patients with COVID-19 about post-discharge thromboprophylaxis, relevant studies may have been published since the end of our search date.

## Conclusions

COVID-19 disease is associated with a hypercoagulable state that has increased VTE risk. Since COVID-19 coagulopathy persists after the acute phase of the disease, extended thromboprophylaxis remains controversial. Based on this systematic review, which included studies and guidelines, after a risk/benefit assessment, post-discharge AC therapy can be reasonable in high-risk patients. Clinical characteristics and laboratory data accompanying RAMs, particularly IMPROVE-DD, can help predict VTE risk. After distinguishing patients who need post-discharge AC therapy, DOACs for 30–35 days and LMWHs for 40–45 days can be the drug of choice. Further studies, particularly the results of the ongoing RCTs, are required to choose better the type of AC, dosage, and duration of prophylaxis. In addition, lifestyle modification is also an aspect to consider when deciding to use AC for post-discharge COVID-19 patients.

### Supplementary Information

**Additional file1**. **Appendix A.1**. Search strategy. **Appendix A.2**. Risk of bias assessment of included studies based on Newcastle-Ottawa Scale (NOS), adopted NOS, and Jadad scale.

## Data Availability

Not applicable.

## Abbreviations

COVID-19: Coronavirus disease 2019
VTE: Venous thromboembolism
PE: Pulmonary embolism
DVT: Deep vein thrombosis
ICU: Intensive care unit
AC: Anticoagulant
RCT: Randomized controlled trial
PRISMA: Preferred reporting items for systematic reviews and meta-analyses
NOS: Newcastle–ottawa scale
AHRQ: Agency for healthcare research and quality
OR: Odds ratio
CI: Confidence interval
RR: Risk ratio
RAM: Risk assessment model
IMPROVE: The international medical prevention registry on venous thromboembolism
CCU: Cardiac care unit
PPS: Padua prediction score
DOAC: Direct oral anticoagulants
LMWH: Low molecular weight heparins
UFH: Unfractionated heparin
CRP: C-reactive protein
CKD: Chronic kidney disease
ACR: Albumin/creatinine ratio
IQR: Interquartile range
n/a: Not available
DTI: Direct thrombin inhibitor
BMI: Body mass index
