# Supplementary material for: A systematic review on post-discharge venous thromboembolism prophylaxis in patients with COVID-19
Source: Egypt Heart J. 2023 Aug 18;75:72. doi: 10.1186/s43044-023-00400-2 (PMC10439090; doi:10.1186/s43044-023-00400-2)
Supplement: Supplementary file 1 — Additional file1. Appendix A.1. Search strategy. Appendix A.2. Risk of bias assessment of included studies based on Newcastle-Ottawa Scale (NOS), adopted NOS, and Jadad scale. [file 43044_2023_400_MOESM1_ESM.docx]

# Appendix A.1. Search strategy

| Databases | Search query |
| --- | --- |
|  |  |
| PubMed, EMBASE, Web of Science, Scopus, Cochrane, and clinicaltrials.gov | (covid OR coronavirus OR 'sars-cov-2') AND ('thromboembolic' OR 'thromboembolism' OR pe OR 'pulmonary embolism' OR 'lung embolism' OR 'deep vein thrombosis' OR dvt OR 'venous thromboembolism' OR vte) AND ('post-discharge' OR 'postdischarge' OR 'after discharge' OR 'hospital discharge' OR 'thromboprophylaxis' OR 'extended') |

# Appendix A.2. Risk of bias assessment of included studies based on Newcastle-Ottawa Scale (NOS), adopted NOS, and Jadad scale.

| Authors (reference), Year | Quality Assessment Tool | | | | | | | | Total Score | Study Quality |
| --- | --- | --- | --- | --- | --- | --- | --- | --- | --- | --- |
|  | **NOS for Cohort Studies ^a^** | | | | | | | |  | |
|  | Question 1 | Question 2 | Question 3 | Question 4 | Question 5 | Question 6 | Question 7 | Question 8 |  |  |
| Spyropoulos et al. 2022 | **🟑** | **🟑** | **🟑** | **0** | **0** | **🟑** | **🟑** | **🟑** | 6 | Poor ^b^ |
| Vaughn et al. 2022 | **🟑** | **🟑** | **🟑** | **0** | **0** | **🟑** | **🟑** | **0** | 5 | Poor |
| Motloch et al. 2022 | **🟑** | **🟑** | **🟑** | **0** | **🟑🟑** | **🟑** | **🟑** | **🟑** | 8 | Good |
| Courtney et al. 2022 | **🟑** | **🟑** | **🟑** | **0** | **🟑🟑** | **🟑** | **🟑** | **🟑** | 8 | Good |
| Tholin et al. 2021 | **🟑** | **🟑** | **🟑** | **0** | **0** | **🟑** | **🟑** | **0** | 5 | Poor |
| Quiros Ambel et al. 2021 | **🟑** | **🟑** | **🟑** | **0** | **0** | **🟑** | **🟑** | **🟑** | 6 | Poor |
| Engelen et al. 2021 | **🟑** | **🟑** | **🟑** | **0** | **0** | **🟑** | **🟑** | **0** | 6 | Poor |
| Li et al. 2021 | **🟑** | **🟑** | **🟑** | **0** | **🟑** | **🟑** | **🟑** | **🟑** | 7 | Good |
| Tan et al. 2021 | **0** | **0** | **🟑** | **0** | **0** | **🟑** | **🟑** | **0** | 3 | Poor |
| Giannis et al. 2021 | **🟑** | **🟑** | **🟑** | **0** | **0** | **🟑** | **🟑** | **0** | 5 | Poor |
| Tsaplin et al. 2021 | **🟑** | **🟑** | **🟑** | **0** | **0** | **0** | **🟑** | **🟑** | 6 | Poor |
| Eswaran et al. 2020 | **🟑** | **🟑** | **🟑** | **0** | **🟑🟑** | **🟑** | **🟑** | **🟑** | 8 | Good |
| Rashidi et al. 2020 | **🟑** | **🟑** | **🟑** | **0** | **0** | **🟑** | **🟑** | **🟑** | 6 | Poor |
| Salisbury et al. 2020 | **🟑** | **🟑** | **🟑** | **0** | **0** | **🟑** | **🟑** | **0** | 5 | Poor |
| Patell et al. 2020 | **🟑** | **🟑** | **🟑** | **0** | **0** | **🟑** | **🟑** | **0** | 5 | Poor |
|  | **Adopted NOS for Cross-sectional Study ^c^** | | | | | | | |  | |
|  | Question 1 | Question 2 | Question 3 | Question 4 | Question 5 | Question 6 | Question 7 |  |  |  |
| Parks et al. 2022 | **🟑** | **🟑** | **🟑** | **🟑🟑** | **0** | **🟑** | **🟑** |  | 7 | Good ^d^ |
| Stawiarski et al. 2021 | **🟑** | **0** | **0** | **🟑🟑** | **0** | **🟑🟑** | **0** |  | 5 | Satisfactory |
|  | **Jadad Scale for RCT study ^e^** | | | | | | | |  | |
|  | Question 1 | Question 2 | Question 3 |  |  |  |  |  |  |  |
| Ramacciotti et al. 2021 | **🟑🟑** | **🟑** | **🟑** |  |  |  |  |  | 4 | Good ^f^ |

**a. NOS domains for cohort studies:**

**Selection:** 1- Representativeness of the exposed cohort. 2- Selection of the non-exposed cohort. 3- Ascertainment of exposure 4- Demonstration that outcome of interest was not present at start of study; **Comparability:** 5- Comparability of cohorts on the basis of the design or analysis; **Outcome:** 6- Assessment of outcome. 7- Was follow-up long enough for outcomes to occur. 8- Adequacy of follow-up of cohorts.

**b. Grading of NOS for cohort studies**

Thresholds for converting the NOS to AHRQ standards (good, fair, and poor): **Good** quality: 3 or 4 stars in selection domain, 1 or 2 stars in comparability domain AND 2 or 3 stars in outcome/exposure domain; **Fair** quality: 2 stars in selection domain AND 1 or 2 stars in comparability domain AND 2 or 3 stars in outcome/exposure domain; **Poor** quality: 0 or 1 star in selection domain OR 0 stars in comparability domain OR 0 or 1 stars in outcome/exposure domain.

**c. NOS domains for cross-sectional studies:**

**Selection:** 1- Representativeness of the sample. 2- Sample size. 3-Non-respondents. 4- Ascertainment of the exposure (risk factor); **Comparability:** 5- The subjects in different outcome groups are comparable, based on the study design or analysis. Confounding factors are controlled; **Exposure:** 6- Assessment of the outcome. 7- Statistical test.

**d. Grading of adopted NOS for cross-sectional studies**

Thresholds for converting the adopted NOS based on Herzog et al. study (very good, good, satisfactory, and unsatisfactory): **Very Good** Studies: 9-10 stars; **Good** Studies: 7-8 stars; **Satisfactory** Studies: 5-6 stars; **Unsatisfactory** Studies: 0-4 stars.

**e. Jadad scale domains for RCT studies:**

**Randomization**, **Blinding**, and **Withdrawals.**

**f. Grading of Jadad scale for RCT studies:**

Thresholds for converting the Jadad scale based on Falagas et al. study (good and poor): **Good** quality: 2 stars and above; **Poor** quality: 0-1 stars.
